# Assessing Mucoadhesion in Polymer Gels: The Effect of Method Type and Instrument Variables

**DOI:** 10.3390/polym10030254

**Published:** 2018-03-01

**Authors:** Jéssica Bassi da Silva, Sabrina Barbosa de Souza Ferreira, Adriano Valim Reis, Michael Thomas Cook, Marcos Luciano Bruschi

**Affiliations:** 1 Laboratory of Research and Development of Drug Delivery Systems, Postgraduate Program in Pharmaceutical Sciences, Department of Pharmacy, State University of Maringa, Maringa, Parana CEP 87020-900, Brazil; jessicabassidasilva@gmail.com (J.B.d.S.); sbsferreira88@gmail.com (S.B.d.S.F.); avreis77@gmail.com (A.V.R.); 2 Research Centre in Topical Drug Delivery and Toxicology, Department of Pharmacy, Pharmacology and Postgraduate Medicine, University of Hertfordshire, Hatfield AL10 9AB, UK; m.cook5@herts.ac.uk

**Keywords:** pluronic f127, thermoresponsive polymers, thermogelling polymers, detachment force, rheology, texture profile analysis

## Abstract

The process of mucoadhesion has been widely studied using a wide variety of methods, which are influenced by instrumental variables and experiment design, making the comparison between the results of different studies difficult. The aim of this work was to standardize the conditions of the detachment test and the rheological methods of mucoadhesion assessment for semisolids, and introduce a texture profile analysis (TPA) method. A factorial design was developed to suggest standard conditions for performing the detachment force method. To evaluate the method, binary polymeric systems were prepared containing poloxamer 407 and Carbopol 971P^®^, Carbopol 974P^®^, or Noveon^®^ Polycarbophil. The mucoadhesion of systems was evaluated, and the reproducibility of these measurements investigated. This detachment force method was demonstrated to be reproduceable, and gave different adhesion when mucin disk or ex vivo oral mucosa was used. The factorial design demonstrated that all evaluated parameters had an effect on measurements of mucoadhesive force, but the same was not observed for the work of adhesion. It was suggested that the work of adhesion is a more appropriate metric for evaluating mucoadhesion. Oscillatory rheology was more capable of investigating adhesive interactions than flow rheology. TPA method was demonstrated to be reproducible and can evaluate the adhesiveness interaction parameter. This investigation demonstrates the need for standardized methods to evaluate mucoadhesion and makes suggestions for a standard study design.

## 1. Introduction

Mucosal surfaces cover the nasal, ocular, buccal, rectal, vaginal, and gastrointestinal areas among other parts of the body. Drugs may be administered to these sites for local effect, and their high permeability makes them attractive for systemic drug delivery. However, the natural clearance mechanisms from these sites limit residence time, decreasing drug absorption or duration of local effect [1]. In order to overcome these disadvantages, “mucoadhesive” systems have been developed, which adhere to mucosal membranes through a variety of attractive physicochemical interactions, enhancing retention, and thus the efficacy of medicines [2,3,4,5].

Mucoadhesive polymers are a group of materials employed in different pharmaceutical systems. They are defined as hydrophilic macromolecules, which contain numerous functional organic groups (i.e., carboxylic, hydroxyl, amide, and amine groups) able to establish interactions with mucosal membranes [6,7]. These polymers can be classified according to their interactions with the mucosa (covalent bonds or non-covalent intermolecular interactions). Non-covalent bonds believed to enhance mucoadhesion include hydrogen-bonding, hydrophobic interactions, and electrostatic interactions. Mucoadhesive polymers may be cationic, anionic, or non-ionic [8,9,10]. Anionic polymers, such as poly(acrylic acid) derivates, are believed to form hydrogen bonds below their pKa between their carboxylic groups and the hydroxyl groups of the mucus glycoprotein. It has also been suggested that ion-dipole interactions may occur when in the carboxylate form [9]. Moreover, poly(acrylic acid) derivates may be combined in solution with thermoresponsive polymers, like poloxamer 407 (P407), to enhance retention [11]. Thermoresponsive polymers transition from a liquid to a viscous gel above a critical temperature, allowing for passage through an applicator before thickening upon application to the body [11,12,13].

In vitro or ex vivo techniques are crucial in the performance testing of mucoadhesive drug delivery systems and are cost-effective in selecting efficient systems when compared with in vivo methods. These methods are able to evaluate mucoadhesive formulations, without using animal models, and may offer mechanistic understanding of mucoadhesion [14,15,16,17]. Numerous techniques have been developed to assess and understand the mucoadhesion of drug delivery systems. The development of new methods should be validated by comparison with a gold standard in vitro technique, or in vivo performance. New methods to investigate the mucoadhesive profile of semisolid polymer systems are typically developed in-house on bespoke equipment, and have not been through validation, which emphasizes the importance of standardized techniques [18,19,20]. Furthermore, each dosage form may require different experimental conditions and comparison may only be possible within dosage form types. The detachment force method (also known as the tensile method) is the most widely employed method to investigate adhesive interactions between a mucosal membrane (or other substrate) and a formulation. This method can be used for solid [19,20,21] and semisolid dosage forms [2,4,5,12,19,21,22,23] and it is known that instrumental parameters and experiment design influence test results. Other techniques, such as the rheological method, can result in different responses and interpretations depending on the analysis type used (flow or oscillatory) [19]. Therefore, it is very important to understand the variables of the method for mucoadhesion testing, considering that standardized methods have been required [1,3].

Therefore, this work aimed to investigate the importance of standardizing the conditions to perform the detachment force and the rheological methods for assessing mucoadhesion of semisolid systems, as well as for assessing mucoadhesive interactions by texture profile analysis.

## 2. Materials and Methods

### 2.1. Materials

Carbopol 971P^®^ (C971P), Carbopol 974P^®^ (C974P), and Noveon^®^ Polycarbophil (PCB) were kindly donated by Lubrizol (Sao Paulo, Brazil). Triethanolamine, poloxamer 407 (P407), and mucin from porcine stomach (type II) were received from Sigma-Aldrich (Sao Paulo, Brazil). Porcine oral mucosa was sourced from a local slaughterhouse (Maringa, Brazil) and kept frozen at −20 °C. All reagents were used without further purification.

### 2.2. Preparation of Polymeric Systems

Monopolymeric formulations were prepared using P407 (15% or 20%, *w*/*w*) or C971P, C974P, and PCB (0.10%, 0.15%, 0.20%, 0.25%, or 0.50%, *w*/*w*). P407 solutions were prepared by dispersing the required amount of the polymer in purified water at 5 °C under mechanical stirring (500 rpm). To prepare the poly(acrylic acid)-containing formulations, the required amount mass of the polymer was dispersed in purified water with mechanical stirring.

Binary polymeric blends were prepared by dispersion of C971P, C974P, or PCB (0.10%, 0.15%, 0.20%, 0.25%, or 0.50%, *w*/*w*) in purified water under mechanical stirring. The required amount of P407 (15% or 20%, *w*/*w*) was then added to the preparation. This mixture was maintained for 12 h at the temperature of 4 °C. After this period of time, the preparations were stirred, neutralized using triethanolamine, and refrigerated (4 °C) for at least 24 h before analysis.

### 2.3. Mucoadhesive Analysis by Detachment Force Evaluation

A TA-XTplus texture analyzer (Stable Micro Systems, Surrey, UK) was utilized to investigate the adhesive properties of the formulations [24]. Mucin disks were prepared by the compression of crude porcine mucin (200 mg) using a ring press with a 13-mm diameter die and a compression force of 10 tonnes, applied for 30 s. Moreover, pig buccal mucosa samples were obtained from pigs (white, young, and recent sacrificed) originated from a local slaughterhouse (authorized by the Brazilian Ministry of Agriculture for human consumption). They were cleaned with phosphate saline buffer (PSB), the cheek mucosa was gently removed, and the samples were prepared with the same diameter and area of the mucin disks (132.73 mm^2^), using a surgical scalpel. Samples displaying wounds or bruises were not used. The mucosal substrate (disk or tissue) was then horizontally attached to the lower end of the probe (cylindrical, P/6), using double sided adhesive tape. Prior to testing, the disk or the mucosal tissue was hydrated by submersion in a 5% (*w*/*v*) aqueous solution of mucin or in PSB for 30 s, respectively. The excess surface liquid was withdrawn by gentle blotting. Samples of each formulation (5.0 g) were packed into shallow cylindrical vessels with 20 mm diameter, maintained at 37 °C, and placed under the probe, which was lowered at a speed of 1 mm/s until it reached the mucoadhesive hydrogel surface. Immediately, a downward force of 0.03 N was applied and the probe remained on the surface of the sample for 30 s; then the probe was withdrawn at a rate of 10.0 mm/s until complete detachment of the sample from the mucosal substrate. The Texture Exponent 32 software (Stable Micro Systems, Surrey, UK) was used to determine the force required for the detachment (F_adh_) and the work of adhesion (W_adh_) (the area under the force/distance curve). All measurements were performed with at least six replicates.

Mucin disks and porcine buccal mucosa were chosen as substrate models, due to the great use of them in the literature. Moreover, pigs have a greater similarity of anatomy, physiology, metabolism and histology than other animals when compared with humans [25,26]. Detachment force, the maximum force necessary to remove the sample from mucosal substrate, and work of adhesion, the area under the force-displacement curve, were evaluated by two-way analysis of variance (ANOVA), using Tukey post hoc test. The *p*-value < 0.05 was taken to denote significance.

To evaluate the effects of the instrumental parameters on the F_adh_ and W_adh_, a polymeric system composed of 15% (*w*/*w*) P407 and 0.25% (*w*/*w*) PCB at 37 °C was also studied. A full factorial design 2^4^ + 4C was created by Statistic 8.0^®^ software (StatSoft Company, Tulsa, OK, USA). The influence of the variables: substrate (*X*_1_), force (*X*_2_), speed of upward probe (*X*_3_) and time of substrate-sample contact (*X*_4_) were investigated. Each factor was set at one of two levels, low (−) or high (+) (Table 1). Four central points were also used to evaluate the curvature and the errors related with isolated effects or the interaction between them.

### 2.4. Mucoadhesive Analysis Using Rheological Methods

#### 2.4.1. Continuous Shear (Flow) Rheology

The increase in consistency index due to a synergism between mucin and polymer systems was measured using a modified version of the method described by Hassan and Gallo [27,28]. The polymeric blends previously prepared, 5% (*w*/*w*) mucin aqueous solution (prepared just before the measurements), and their mixture were evaluated. The mixtures were composed of the polymeric systems with 5% (*w*/*w*) mucin added under vigorous stirring for 15 min prior to analysis. Flow rheometry was performed using a controlled stress rheometer (MARS II, Haake Thermo Fisher Scientific Inc., Newington, Germany), equipped with parallel steel cone-plate geometry of 60 mm; separated by a fixed distance of 0.052 mm. Analysis was performed in flow mode at 37 ± 1 °C, over shear rates ranging from 0 to 2000 s^−1^, increasing over a period of 150 s, and was maintained at the superior limit for 10 s and then decreased during the same period. At least six replicates of each sample were evaluated, and the upward flow curves were fitted using Power Law equation (Oswald-de-Waele) as shown below [2,27,29,30]

τ = *k*. γ^*n*^ (1)

where τ is the shear stress (Pa), *k* is the consistency index [(*Pa*.*s*)^*n*^], γ is the rate of shear (s^−1^), and *n* is the flow behavior index (dimensionless).

#### 2.4.2. Oscillatory Rheology

Oscillatory measurements were performed at 37 ± 1 °C, using the same rheometer and geometry previously described, and in oscillation mode. Samples of each polymeric system, mucin aqueous solution 5% (*w*/*w*) and the polymeric systems with 5% (*w*/*w*) mucin were placed to the inferior plate, allowing for 1 min equilibration prior to testing. The linear viscoelastic region of each formulation was determined and a frequency sweep was performed from 0.1 to 10.0 Hz. The elastic modulus (*G*′), viscous modulus (*G*′′), dynamic viscosity (*η*′), and the loss tangent (tan δ) were calculated using RheoWin 4.10.0000 (Haake) software (Thermo Fisher Scientific Inc., Newington, Germany). All measurements were performed in at least six replicates [29]. Calculation of the interaction parameter for the polymeric systems (P407/C971P, P407/C974P, and P407/PCB) with mucin was determined by the difference between the storage (elastic) modulus of the mixture, and the theoretical value of the storage modulus obtained by summation of the individual parts [29,31], at an oscillatory frequency of 10.0 Hz as demonstrated in the equation.

(2) $$\Delta G^{\prime }=G_{\mathrm{mixture}}^{\prime }-\left(G_{\mathrm{mucin}}^{\prime }+G_{\mathrm{polymeric}\;\mathrm{system}}^{\prime }\right)$$

### 2.5. Texture Profile Analysis

The texture profile analysis (TPA) of polymeric blends, 5% (*w*/*w*) mucin aqueous solution and the mixture of both were performed using a TA-XTplus Texture Analyzer (Stable Micro Systems; Surrey, UK) in TPA mode, at 37 °C, to evaluate adhesive interactions. Bottles containing 16 g of formulations were submitted to a double compression by an analytical probe (10 mm diameter). The two times of compression (at 2 mm/s) were performed on the sample, with a predefined depth (15 mm). A delay period of 15 s was permitted between the end of the first compression and the beginning of the second. From the force-time and force-distance plot, the mechanical properties were obtained, namely: hardness (maximum force obtained during the first compression), compressibility (the work required to deform the sample during the first pass of the probe), adhesiveness (work required to overcome the attractive forces between the surfaces of the probe and the formulation), elasticity (ability to stretch and return to its original size and shape), and cohesiveness (work spent to unite the surface of the sample and the surface of the probe) [2,29]. All analysis was performed in at least five replicates. The interaction parameter of these variables was calculated by the difference between the values observed for the mixture (polymeric blend with 5% (*w*/*w*) mucin) and the sum of the individual contributions of polymeric system and mucin solution.

### 2.6. Statistical Analysis

The responses obtained were statistically compared using two-way analysis of variance (ANOVA). In all cases, individual differences between means were identified using Tukey’s test. Moreover, significant differences were accepted when *p* <0.05 for all methods [32].

## 3. Results and Discussion

### 3.1. Polymeric Systems

Previously characterized binary polymer blends [23,30,33,34] were selected to evaluate the robustness of the mucoadhesive methods. These systems, composed of P407 and poly(acrylic acid) derivatives possess different rheological and mechanical properties. These polymers contribute in a unique way to the formulation properties. With a temperature increase, P407 increases the viscosity of the system and the other decreases it (C971P, C974P, or PCB). Although other polymers as natural polymers (i.e., gelatin and agar) and synthetic polymers (i.e., poly(vinylpyrrolidone) and poly(vinyl alcohol)) were already widely used to investigate the mucoadhesion process, the selected thermorresponsive blends, besides being very well characterized as a complex system, can be a challenge for different mucoadhesion analyses [23,30,33,34]. The carbomers C971P and C974P are mucoadhesive polymers crosslinked with allyl-ethers of pentaerythritol, displaying a concentration ranging from 56% to 68% of carboxylic groups in the chain. However, C974P displays a higher crosslinking degree than C971P. PCB is also a high-molecular-weight polymer of polyacrylic acid, cross-linked with divinylglycol or polyalkenyl ethers, displaying a large number of carboxylic groups (COOH) on the molecular chain, and known by its strong mucoadhesive properties [23,30,34]. The P407 monopolymeric formulation displays higher consistency at body temperatures than the blends containing the same amount of this thermogelling polymer [12,23,35]. Rheological, texture, and mucoadhesion studies have been performed to select the most suitable mucoadhesive semi-solid systems of each blend in previous studies (Table 2).

The interaction parameter evaluates the adhesive interaction between polymers and can select optimal formulations for pharmaceutical and biomedical application [29,30]. The gelation temperature and adhesiveness are also important for performance testing of thermogelling systems to ensure that they solidify on application and are retained on the mucosa. The P407/C971P (20/0.20%, *w*/*w*), P407/C974P (15/0.25%, *w*/*w*), and P407/PCB (15/0.25%, *w*/*w*) system were selected based on previously-published information [23,30,34]. A gelation temperature range near the body temperature, between 25 and 37 °C, is considered appropriate for pharmaceutical systems [2,13].

Methods for analyzing the performance (e.g., mucoadhesion) should be investigated for accuracy, precision, specificity, linearity, and range [36]. In this work, some of these characteristics (precision by repeatability and robustness) were studied to evaluate the importance of standardizing the conditions of method. The robustness of an analytical method is defined by ICH guidelines as a measurement of resistance against small variations of the analytical parameter. Thus, it is possible to verify the robustness of the method by demonstrating its reliability during the normal use [36]. In this study, the methods to investigate the mucoadhesive characteristic were kept constant and the semi-solid formulations were changed to consider the formulations as a parameter or independent variable. Even known the temperature is not the same at 37 °C for different routes, and diseases are able to change the body temperature (e.g., fever), 37 °C was kept through the studied methods. Since most mucosal routes demonstrate this mean temperature. In addition, the selected thermoresponsive systems maintain their physicochemical profile with small variation of temperature (30–38 °C) and gel in this temperature range.

### 3.2. Analysis of the Detachment Force

The detachment force is an in vitro method widely used to analyze the mucoadhesive properties of the most dosage forms. By this method, it is possible to measure the W_adh_, which is based on the area under a force-distance curve obtained during the detachment process, as well as the F_adh_, defined as the maximum force to separate an adhesive surface from a mucous substrate [1,3,30,35,37]. There is not a standard methodology to this determination [37,38], so not only the choice of the technique, but also the choice of membrane (mucus or other mucosal types) and instrument parameters are critical to investigate the mucoadhesive properties of drug delivery systems. The limited use of the human mucosa means that ex vivo animal models are required, but these require validation and an understanding of intra-species variability. Moreover, the process of mucus/mucosa preparation can modify the physicochemical properties and results in an altered structure when compared to a fresh mucosa [37,38]. To begin this process, a standardization of this method using mucin disks and oral porcine mucosa for the three semi-solid systems was proposed.

**Figure 1:**
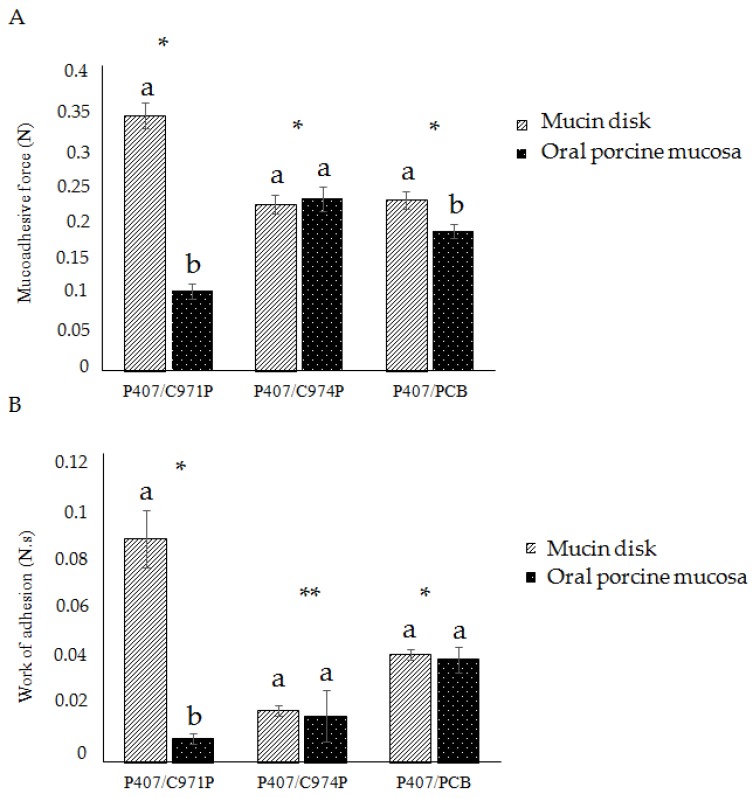
Mucoadhesive force F_adh_ (**A**) and work of adhesion W_adh_ (**B**) of the polymeric blends containing P407 and C971P, C974P, or PCB, obtained by detachment force method using porcine mucin disks or oral porcine mucosa as substrates. The symbols (* and **) and the letters (a and b) represent the significant difference (*p* < 0.05) among the polymeric systems and the substrates, respectively.

The results of the detachment force method (Figure 1 and Table S1) demonstrate the reproducibility of the method with a coefficient of variation (or relative standard deviation), lower than 5% for mucin disks and lower than 12% for porcine oral mucosa, with the variation believed to be a result of the mucosa’s natural variability [33,39,40]. Previous studies have demonstrated differences among mucin from different sites. Likewise, the variability between individuals and mucosa thickness may also affect results on ex vivo mucosa [16,37,41]. In a previous investigation using mucin disks, polymeric blends containing P407/C971P, P407/C974P, or P407/PCB showed the same values of the mucoadhesion force, when compared by this method, supporting its reliability between laboratories [23,30,34].

The P407/C971P formulation demonstrated the greatest mucoadhesive strength on mucin disks, with the rank order P407/C971P > P407/PCB > P407/C974P. Binary polymeric systems composed of P407 and poly(acrylic acid) derivatives have the availability of the carboxylic groups present in the mucoadhesive polymer decreased. It is believed that interactions between P407 and the mucoadhesive polymer reduces the possibility of interaction between the poly(acrylic acid) derivate and the mucin. However, C971P has higher carboxyl concentration, when compared with C974P and PCB, which allows the P407/C971P system to interact with mucin groups even with the P407 interaction [2,33,34,39,42,43]. Moreover, P407/C971P demonstrated in TPA analysis higher values of hardness, cohesiveness, and compressibility, which contribute with the higher values of mucoadhesion observed with mucin disk. Using porcine buccal mucosa as a substrate gave a different rank-order of mucoadhesion: P407/C974P > P407/PCB > P407/C971P. The PCB with an intermediate amount of free carboxyl groups had in the two cases, also the intermediate mucoadhesive force. Furthermore, C974P, a more cross-linked material with a lower amount of hydroxyl groups, demonstrated similar results using mucin disk and animal mucosa. It is believed that the polymer has greater attractive forces among its polymeric chains, which can be also observed ahead by the high cohesiveness value, in TPA analysis. Creating a more cohesive system, with reduced mucin interaction, macroscopic differences were not observed with the substrate variation [19,23,44].

The discrepant answers observed between the results obtained with the two substrates for the P407/C971P system can suggest this formulation uses hydration to establish adhesive bonds between mucin and polymeric blend [1,40,45]. The C971P polymer exhibits lower cross-linking degree allowing higher swelling in presence of water. Therefore, in a low-hydration surface, such as the mucin disk, this system does not swell and displays a greater response than in high hydrated surface as the animal mucosa [43]. The water concentration also influences the tensile strength method [42], and a super hydration state of some polymers can reduce their mucoadhesive performance [43,46,47,48]. Thus, the selection of the mucosal surface needs to consider the composition and consistency of the formulation analyzed, where the most consistent systems (with high mechanical resistance) have better response using mucin disks.

To compare in vitro and in vivo tests, the same condition of analysis should be used [17]. However, using the same parameters, significant differences were observed between analyses performed with isolated mucin and ex vivo mucosa, particularly in the P407/C971P system. Furthermore, the W_adh_ response, related to elasticity and plasticity, demonstrated a significant difference between the three polymeric systems. On the other hand, for the F_adh_ response, significant difference was not found between the binary blends (*p* > 0.05). Therefore, the W_adh_ is suggested as a better response for the detachment force method and, when calculated as an N·mm unit, it can be converted to Joules, showing the energy dispending at the separation process of the adhesive surfaces.

In order to optimize this method, the responses of the P407/PCB polymeric blend were evaluated (Table 3) under the variation of the instrumental parameters in a factorial design.

**Figure 2:**
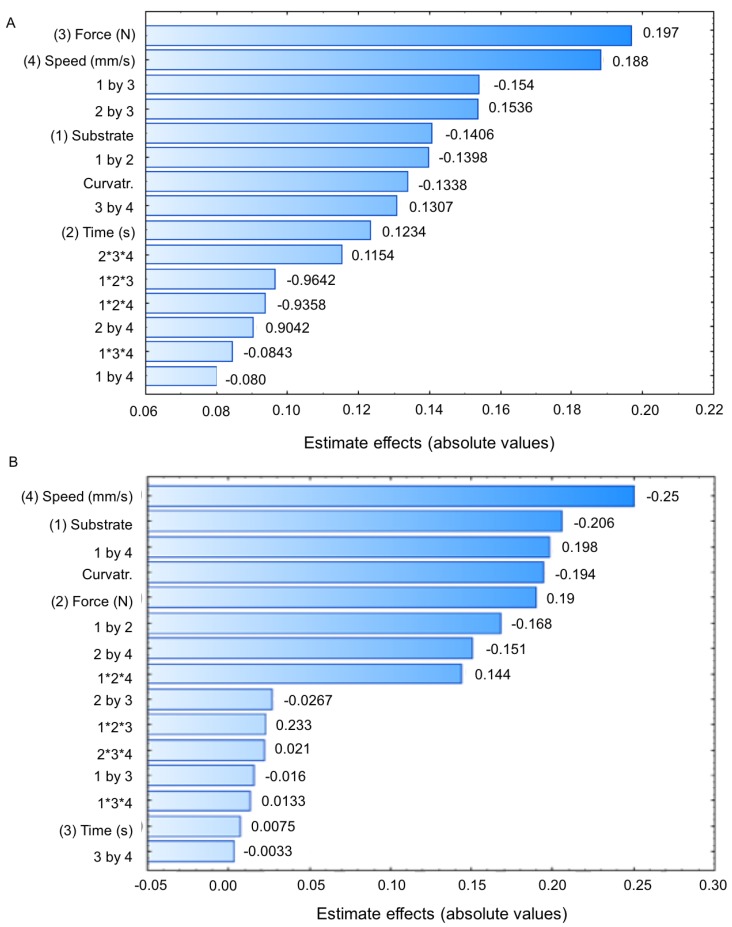
Pareto diagrams with the estimate effect of the parameters about the mucoadhesive force F_adh_ (**A**) and work adhesion W_adh_ (**B**) for the binary polymeric system composed of 15% (*w*/*w*) P407 and 0.25% (*w*/*w*) PCB, at 37 °C, and their interactions.

The Pareto diagrams (Figure 2) shows the estimated effect of each factor and their interactions. A factor and an interaction are considered to influence the response only if the estimated effect is significant, i.e., *p* < 0.05. It was found that variation of substrate, force, probe velocity rate, and contact time influenced the F_adh_. Applied force had the highest positive influence, and the substrate had a negative influence on this response of mucoadhesive force. For the W_adh_, only the contact time and some interactions demonstrated positive influence on the response. Speed and the change of the substrate from mucin disk to animal mucosa had the greatest negative contribution.

**Figure 3:**
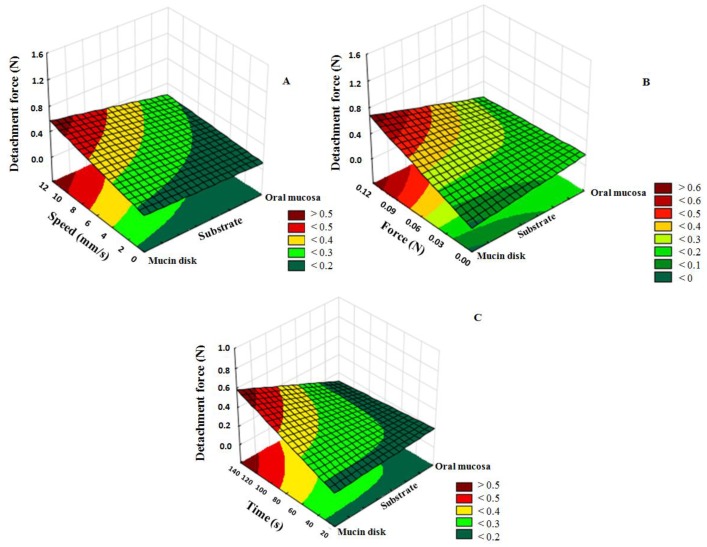
Response surface plots of the detachment force (F_adh_) using mucin disk or oral mucosa influenced by some factors: (**A**) speed of probe ascent (mm/s); (**B**) applied force (N); (**C**) analysis time (s).

**Figure 4:**
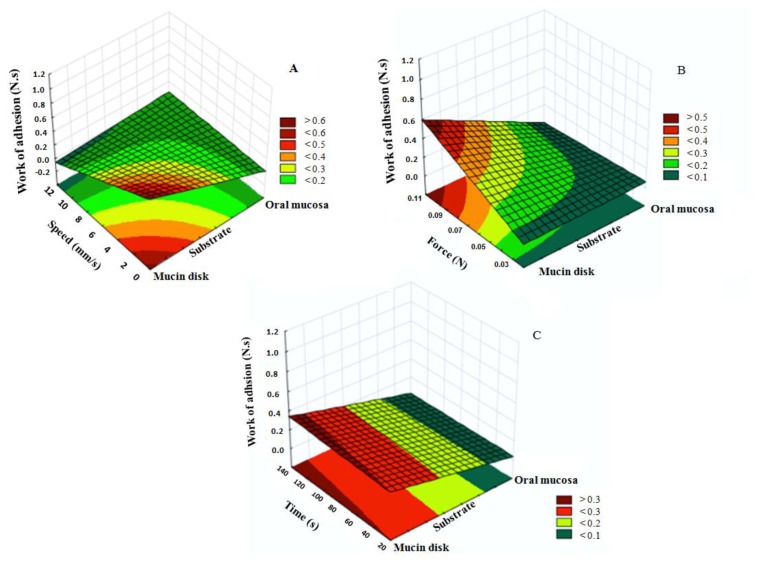
Response surface graphs of the work adhesion (W_adh_) using mucin disk or oral mucosa influenced by some factors: (**A**) speed of probe ascent (mm/s); (**B**) applied force (N); (**C**) analysis time (s).

The surface response plots (Figure 3) demonstrate that the largest F_adh_ was obtained using the mucin disk, 0.1 N of contact force, 120 s of contact time, and a speed of 10 mm/s. On the other hand, for the W_adh_ (Figure 4), the highest value was observed using mucin disk, 0.1 N, 1 mm/s, which was time independent. The surface was adjusted to the experimental data by multiple adjusted correlation coefficient values *R*^2^_adj_. Thus, through the regression analysis, it was observed that 93.98% of the variation in the F_adh_ response is explained with this model (*R*^2^_adj_ = 0.9398). Moreover, 96.08% of the variation in the W_adh_ response is explained with this model (*R*^2^_adj_ = 0.9608). Therefore, the method can estimate the F_adh_ and the W_adh_ according to these parameters.

**Figure 5:**
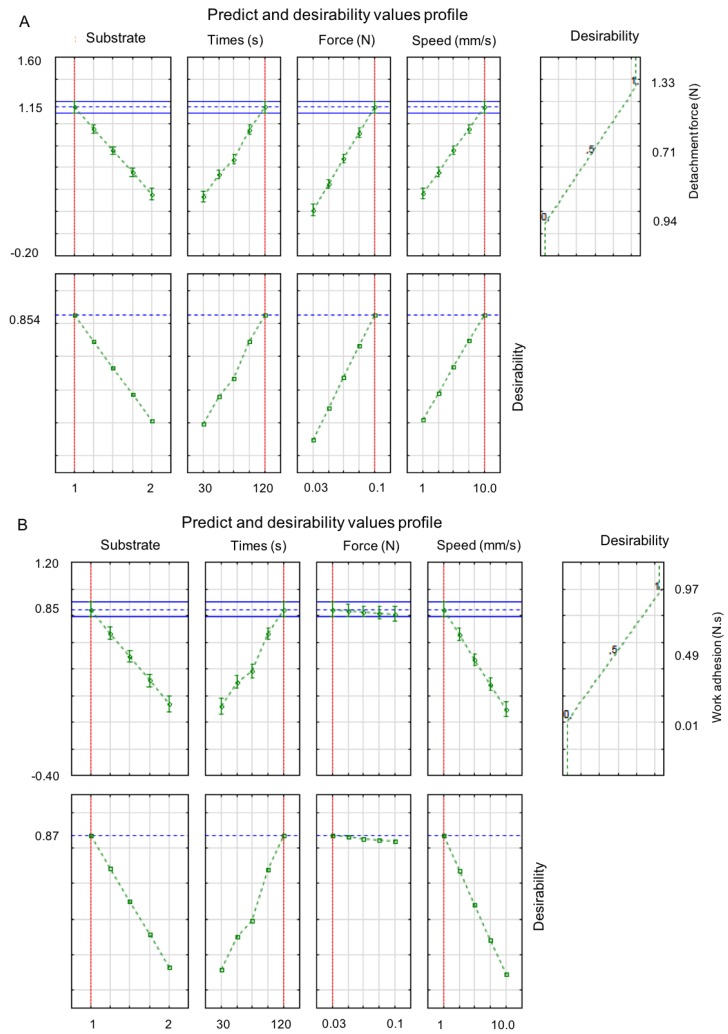
Profile of prediction and desirability values of the detachment force (**A**) and work adhesion (**B**) related to the substrate, time (s), applied force (N), and speed of probe ascent (mm/s).

Additionally, the desirability of the method was evaluated for both responses, which represents the combination of factors required to obtain a better response [49]. Figure 5 shows the desirability response for the F_adh_ results, which is 85% of the maximum value experimentally obtained (1.147 N).

About the W_adh_ (Figure 5B), the desirability was at 92% of the maximum experimental value (1.019 N.mm). Moreover, the substrate variation from the mucin disk to the animal mucosa reduced both results for the studied semi-solid formulation. It was also observed that the F_adh_ increases when changing the levels from minimum to maximum for time, force, and speed parameters. Prior studies had demonstrated the increase of contact time between formulation and substrate surface increases the interaction between mucin and polymer chains; thus, higher values of adhesion are obtained [43,50]. About the W_adh_, changing the levels from minimum to maximal for force and speed parameters, the response is greater as well. On the other hand, the time was not relevant to this answer. The statistical analysis suggests that it is not possible to compare results present in the literature, which use different substrates and parameters, for the tensile strength method. Thus, it is clear the necessity of standardization of this method, to have real comparisons among mucoadhesive profiles of different formulations and polymeric systems.

The desirability cannot show the proximity to real values, which characterizes a limitation of the in vitro methods. Nevertheless, this analysis allows a standardization of this method using both responses, since the higher value is obtained using these parameters. Therefore, for semi-solid formulations containing P407 and poly(acrylic) derivate, it can be suggested that the use of mucin disk, a force of 0.1 N, 120 s of contact time, and 10 mm/s probe speed will obtain better F_adh_ measurement. Using a mucin disk, 0.1 N of force, 30 s of contact time, and a probe speed of 10 mm/s can be used to obtain a better W_adh_ response.

Therefore, with the information obtained, it will be possible in future studies to select and validate the best conditions to perform comparisons.

### 3.3. Rheological Methods

Rheological analysis represents an in vitro model which can predict the in vivo behavior of adhesive formulations and also investigate their structural interactions. The mucoadhesion process is a phenomenon which combines different interaction types and the rheological method is used to evaluate these interactions between mucin and polymeric system [3,16,18,51]. The mucoadhesion process is characterized when the rheological response of a polymer-mucin mixture is higher than the isolated contributions, giving rise to an “interaction parameter” [8,20,28,47].

There are different ways to use the rheological analysis in studying mucoadhesion. It can be performed by consistency measurement over a range of shear rates or by monitoring the viscoelastic properties [27]. Whilst rheology is preferred as a secondary technique, it is often used to measure changes in viscosity or elastic behavior [2,8,23,40,47]. However, a lack of understanding about the meaning of the interaction parameter further confounds the great variability of results in the literature. Therefore, a standardization of the interpretation of results from the rheological analysis was proposed for the three semi-solid systems using the continuous shear analyses to evaluate the mucoadhesive properties (Table 4).

The addition of mucin to the polymeric system demonstrates reduced consistency of the P407/C971P and P407/C974P systems, with a positive interaction parameter for the PCB-containing system. Consistency is a measure of internal friction and the presence of mucin appears to reduce interaction within the formulation. Comparing with the data in Figure 1A, it can be seen that P407/C971P had the greatest force of adhesion, and a very large, negative, interaction parameter. It is suggested that a negative parameter for the consistency index could be a result of interaction between mucin and mucoadhesive polymer, which reduces bridging of poloxamer micelles in the formulation, lowering the overall consistency. It is also possible that the mucin glycoproteins affect the micellization behavior in an unpredictable manner. On the other hand, the P407/PCB polymeric system mixed with mucin exhibited a consistency index greater than the mucin and polymeric systems isolated. This is evidence of a strong interaction between the polymers and mucin, forming a highly cohesive system.

As already described in the literature, there are disadvantages in the use of flow rheometry as a method to evaluate mucoadhesive properties. The continuous shear analysis is a destructive test, then the mucin–polymer interactions can be disrupted, and it is not often possible to observe the mucoadhesion phenomenon [8,29,40,47]. This study exposes the limitations of the continuous flow rheological method to evaluate the mucoadhesive profile of semi-solid systems, and it is best used to understand mechanistic aspects.

For oscillatory rheology, the synergism between polymer and mucin is employed to evaluate mucoadhesion. This technique has advantageous characteristics, such as being a non-destructive method which simulates the formulation behavior during application. Recently, Jones and co-workers [52] reinforced the correlation between viscoelasticity and mucoadhesion, using linear regression [8]. The adhesive interactions in the polymeric blends were observed by the elastic modulus (*G*′) analysis of the mixture as a function of frequency. When the *G*’ of the mixture is higher than the isolated polymer and mucin contributions, ∆*G*′ > 0, rheological synergism occurs [2,29,53]. The observed and calculated values of *G*′ moduli were obtained at 10.0 Hz, since in this oscillatory frequency the systems are forced at higher oscillatory intensity.

The elastic, or “storage” modulus measures the storage and recovered energy at each deformation cycle, reflecting the solid component of a viscoelastic material [47]. The elastic modulus will demonstrate higher values if a sample is predominantly elastic, i.e., highly structured. In contrast, the loss modulus (*G*′′) demonstrates the lost energy at each cycle, and will be higher when the sample is predominantly viscous. The frequency sweep at the linear viscoelastic region allows the three-dimensional structure of the sample to be preserved throughout analysis. The type of cross-linked structure can be revealed in the oscillations, where a small effort is exerted at each frequency. The oscillatory analysis allows the differentiation of the physical entanglements and secondary bonds, since in low frequencies the polymeric physical entanglements can be separated, while, secondary bonds remain fixed [46,47]. The results are displayed in Table 5.

The viscoelastic response of the systems suggests the entanglement and secondary bonds (hydrogen bonds) between mucoadhesive polymers and mucus glycoproteins for the P407/C974P and P407/PCB binary systems, since the establishment of secondary bonds results in the increase of the elasticity into the formulations. Furthermore, the difference observed on the rheological interaction parameter of the three formulations can be attributed to the structural differences of these polymers [46,54].

Formulations which show high viscosity have demonstrated to suffer low clearance and, consequently, they remain longer at the action site [55]. However, despite having higher viscosity, the P407/C971P platform displayed rheological antagonism, demonstrating that the addition of mucin did not increase overall interaction in the system. Edsman & Hägerström had already demonstrated positive values (rheological synergism) for low concentrations of cross-linking polymers, while high concentration of them results in negative values. In this sense, the evaluation of the *G*’ gives positive values for weakly hydrogels and strongly cross-linked hydrogels will demonstrate a negative interaction parameter [20].

The system composed of P407 and C971P has the higher polymeric concentration when compared with the P407/C974P and P407/PCB formulations. Large concentrations of the polymers into the mixture added mucin, as well as the high viscosity of this system reduces the availability of the solvent, which makes the interpenetration of the polymer and mucin chains difficult, since them flexibility and mobility are reduced [47,56].

Oscillatory rheometry is a valid and sensitive method to evaluate interactions in polymer-mucin mixtures, and although the P407/C974P and P407/PCB formulations had demonstrated similar answers between these methods, a direct comparison with the standard tensile method was not possible. Moreover, it was observed that the results depend more on the polymer concentration than of the polymers chemical structure, since P407/C974P and P407/PCB systems exhibited similar results. In this way, to use the oscillatory rheology as a method to evaluate the mucoadhesive profile of semi-solid formulations, a prior cohesiveness analyses must be done.

### 3.4. Texture Profile Analysis

TPA is a quick and common analytical methodology. It can be used for the mechanical characterization of semi-solid pharmaceutical dosage forms and aid understanding of structure. The results of this analysis allow easy identification of the physicochemical interactions among the components of the formulations and also allow prediction of the behavior of these systems under different analytical conditions, and during use in pharmaceuticals. The values obtained by this technique are: hardness, elasticity, adhesiveness, cohesiveness, and compressibility [57,58].

The development of pharmaceutical dosage forms for topical application requires formation of a target profile. These formulations need to be easily removed from the packing to have good spreadability, bio/mucoadhesion, and adequate viscosity in order to facilitate retention and thus patient compliance with the treatment. Moreover, for mucoadhesive systems the resistance to the natural defense processes needs to be considered [34,40,57,58].

To be easily applied, a semi-solid needs to demonstrate low values for hardness and compressibility, since these values indicate how easy it is to remove the formulation from the packing, as well as the spreadability and removal of the product at the desirable site. However, very low values of them can impair the retention of the formulation. Ideally, semi-solid systems must demonstrate high values of adhesion to ensure retention. In addition, high values of elasticity aid retention, because the systems have a tendency to return to their structure. On the other hand, high values to the cohesiveness parameter are also desirable [2,23,29,57,58,59,60].

In order to obtain pharmaceutical systems with acceptable mechanical characteristics, ensuring in vivo retention and therapeutic efficiency, the texture profile properties of the formulations must be studied. As previously mentioned, the chosen systems have already demonstrated themselves to be favorable pharmaceutical systems, about the TPA properties [23,30,34]. In addition to studying mechanical properties, it may be used to evaluate the mucoadhesion profile of semi-solid systems by the analysis of the polymer-mucin interaction.

Comparing the results of polymeric systems with and without mucin (Table 6) there was a decrease in the hardness, compressibility and adhesiveness. The cohesiveness had increased values for two of the three systems, and the elasticity was unaltered. The P407/C971P system demonstrated higher values of hardness, compressibility and adhesiveness than P407/C974P and P407/PCB, probably because its polymeric concentration is also greater. In this sense, this formulation displayed also superior hardness, compressibility and adhesiveness responses. However, the decrease of this parameter after mucin addition was more significant, since the C971P presents a lower degree of crosslinking; therefore, it has a greater number of free carboxyl groups for interaction with the mucin, which promotes a more intense disintegration of this blend when compared with those containing other adhesive polymers (C974P and PCB). Similar to rheological analysis, it appears that polymer–mucin interactions may not provide constructive synergism in these systems.

The absence of change in the elasticity of the systems, even with the mucin addition, reflects the results obtained in the oscillatory rheology where the viscoelastic property was retained. Furthermore, the decrease of hardness and compressibility in the added mucin systems agrees with the flow rheology and demonstrates the loss of internal friction in these systems after mucin addition. The cohesiveness of the P407/C974P blend with mucin was superior to the two other blends. Probably, it is due to the C974P chemical structure, since it is a poly(acrylic acid) derivative with a higher degree of cross-linking, providing a more cohesive system, and the increase of the attractive force in the formulation is more dramatic.

**Figure 6:**
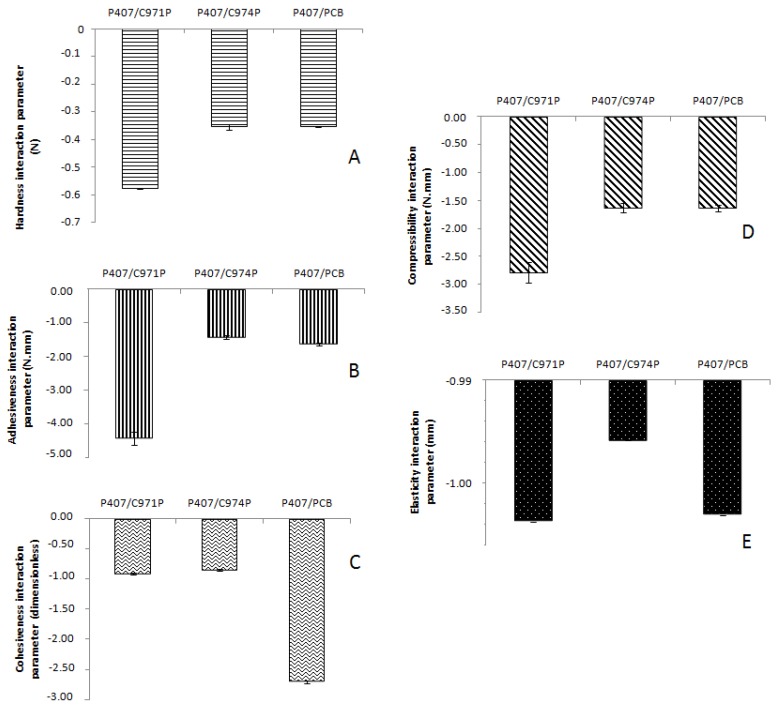
Mechanical properties obtained by texture profile analysis (TPA) of the polymeric systems containing poloxamer 407 (P407), mucin and Carbopol 971P^®^ (C971P), Carbopol 974P^®^ (C974P) or Noveon^®^ Polycarbophil (PCB) and of pure mucin solution: (**A**) hardness, (**B**) adhesiveness, (**C**) cohesiveness, (**D**) compressibility, (**E**) elasticity.

The interaction parameters of the responses obtained by TPA were calculated between the polymeric blends and mucin solution (Figure 6); this is an important value to investigate the mucoadhesive profile of the semi-solid preparations. All polymer blends showed a negative interaction parameter, i.e., the mixture with mucin in all the cases obtained lower values when compared with the pure polymeric systems. In this way, the results suggest the interaction between mucoadhesive polymer and mucin, which promotes mechanical changes.

The adhesiveness interaction parameter is a result of interaction between the formulation and polycarbonate probe. Therefore, negative values for mucoadhesive formulations are expected. Considering a higher adhesive polymer-mucin interaction, there are fewer free carboxyl groups to interact with the probe, and the adhesiveness of the mixture on the probe is lower when compared with the mixture with added mucin. Thus, as a new way to measure mucoadhesive properties of semi-solid systems, the analysis of the adhesiveness interaction parameter can be simple and accessible with fast execution. Moreover, a correlation was observed between this method and the tensile strength method using the mucin disks, since the adhesive profile of the three evaluated systems demonstrated a similar ranking evaluation.

## 4. Conclusions

This study utilized the tensile strength method with porcine mucin disks and porcine oral (cheek) mucosa, flow and oscillatory rheometry, as well as texture profile analysis, to evaluate the mucoadhesive performance of the three polymeric systems composed of poloxamer 407 and poly(acrylic acid) derivatives. It was also possible to investigate these methods to understand the parameters which can influence experimental results, highlighting the need for standardization. The reproducibility of the methods for these semi-solid formulations was also shown. The tensile strength method demonstrated differences when comparing the mucin disk and oral ex vivo mucosa. The factorial design displayed that all evaluated parameters have an effect in the F_adh_; but the same was not observed for W_adh_, for which most interactions did not influence response. W_adh_ was suggested as a more appropriate metric for evaluating mucoadhesion. The oscillatory rheology was more capable of showing adhesive interactions than continuous flow rheology. However, each rheological analysis needs to be associated with complementary analyses. Furthermore, the texture profile analysis method with the mucin addition was shown to be reproducible by the evaluation of the adhesiveness interaction parameter. In this sense, each one of the methods has an important place within the evaluation of mucoadhesion of semi-solid pharmaceutical systems, but it is very important to understand the importance and influence of the conditions of analysis on experimental results. There is a clear need for standardized methods to evaluate mucoadhesive properties of semisolid drug delivery systems.

## Figures and Tables

**Table 1:** Matrix of factorial design 2^4^ + 4C for binary polymeric systems composed of 15% (*w*/*w*) P407 and 0.25% (*w*/*w*) PCB, at 37 °C, and values for the low and high levels of each variable.

Standard Run	Independent Variables			
	*X*_1_	*X*_2_	*X*_3_	*X*_4_
1	A	−	−	−
2	B	−	−	−
3	A	+	−	−
4	B	+	−	−
5	A	−	+	−
6	B	−	+	−
7	A	+	+	−
8	B	+	+	−
9	A	−	−	+
10	B	−	−	+
11	A	+	−	+
12	B	+	−	+
13	A	−	+	+
14	B	−	+	+
15	A	+	+	+
16	B	+	+	+
17	A	0	0	0
18	B	0	0	0
19	A	0	0	0
20	B	0	0	0
Factor		−	0	+
*X*_1_	Substrate	A: Mucin disk		B: Oral pig mucosal
*X*_2_	Force (N)	0.03	0.065	0.10
*X*_3_	Speed (mm/s)	1.00	5.50	10.0
*X*_4_	Time (s)	30.0	75.0	120.0

**Table 2:** Mucoadhesive profile (using porcine mucin disk), gelation temperature, and rheological interaction parameter of the selected formulations containing poloxamer 407 (P407) and Cabopol 971P^®^ (C971P), Carbopol 974P^®^ (C974P), or Noveon^®^ Polycarbophil (PCB).

Systems	Polymer Amount (%, *w*/*w*)	Mucoadhesive Force (N)	Gelation Temperature (°C)	Interaction Parameter (Pa)
P407/C971P ^a^	20/0.20	0.352 ± 0.04	27.88 ± 0.06	1944.73 ± 381.93
P407/C974P ^b^	15/0.25	0.231 ± 0.03	36.04 ± 0.06	2509.33 ± 215.85
P407/PCB ^c^	15/0.15	0.237 ± 0.01	36.42 ± 0.02	1927.03 ± 93.85

^a^ [34]; ^b^ [30]; ^c^ [23].

**Table 3:** Response values of the mucoadhesive force (N) and the work adhesion (N·mm) of the binary polymeric system containing 15% (*w*/*w*) P407 and 0.25% (*w*/*w*) PCB, at 37 °C, using porcine mucin disks (A) or porcine oral mucosa (B). Each value represents the mean of at least three replicates. In all cases, the coefficient of variation of replicate analyses was less than 12%.

Experiment	Factors				Adhesive Force (N)	Work (N·mm)
	Substrate	Applied Force (N)	Velocity (mm/s)	Time (s)		
1	A	0.03	1.00	30	0.133	0.131
2	B	0.03	1.00	30	0.121	0.081
3	A	0.10	1.00	30	0.189	0.829
4	B	0.10	1.00	30	0.128	0.110
5	A	0.03	10.0	30	0.169	0.313
6	B	0.03	10.0	30	0.255	0.110
7	A	0.10	10.0	30	0.329	0.927
8	B	0.10	10.0	30	0.223	0.405
9	A	0.03	1.00	120	0.138	0.259
10	B	0.03	1.00	120	0.120	0.055
11	A	0.10	1.00	120	0.365	0.740
12	B	0.10	1.00	120	0.110	0.083
13	A	0.03	10.0	120	0.200	0.469
14	B	0.03	10.0	120	0.115	0.245
15	A	0.10	10.0	120	1.247	1.033
16	B	0.10	10.0	120	0.303	0.383
17 (C)	A	0.065	5.50	75	0.191	0.548
18 (C)	B	0.065	5.50	75	0.157	0.125
19 (C)	A	0.065	5.50	75	0.236	0.715
20 (C)	B	0.065	5.50	75	0.169	0.242

**Table 4:** Values of the consistency index (*k*) for polymeric blends containing poloxamer 407 (P407) and Carbopol 971P^®^ (C971P), Carbopol 974P^®^ (C974P), or Noveon^®^ Polycarbophil (PCB)^a^ at 37 °C. Each value represents the mean (±standard deviation) of at least six replicates.

Formulation	*k* (Pa.s) of Polymeric System	*k* (Pa.s) of Mixture	Interaction Parameter (Pa.s)
P407/C 971P	43,143.33 ± 470.78	227.70 ± 9.74	−42,915.67
P407/C974P	139.40 ± 6.01	107.60 ± 2.84	−31.83
P407/PCB	60.96 ± 3.31	97.63 ± 2.07	36.64
Mucin solution	0.033 ± 0.001		

**Table 5:** Values of the elastic modulus (*G*′) for polymeric blends containing poloxamer 407 (P407) and Carbopol 971P^®^ (C971P), Carbopol 974P^®^ (C974P), or Noveon^®^ Polycarbophil (PCB)^a^ at 37 °C. Each value represents the mean (±standard deviation) of at least six replicates.

Formulation	*G*′ (Pa) Polymeric System	*G*′ (Pa) Mixture	Interaction Parameter (Pa)
P407/C971P	16,646.00 ± 612.97	15,368.17 ± 865.56	−1305.68
P407/C974P	3246.00 ± 227.49	5528.83 ± 320.13	2254.99
P407/PCB	2660.00 ± 85.46	4826.50 ± 279.20	2138.65
Mucin	27.85 ± 2.74		

**Table 6:** Mechanical results obtained by texture profile analysis from the binary polymeric formulations composed of poloxamer 407 (P407) and Carbopol 971P^®^ (C971P), Carbopol 974P^®^ (C974P), or Noveon^®^ Polycarbophil (PCB) with or in the absence of mucin. Each value represents the mean (±standard deviation) of at least six replicates.

System	TPA Results				
	Hardness (N)	Compressibility (N·mm)	Adhesiveness (N·mm)	Elasticity (mm)	Cohesiveness (Dimensionless)
P407/C971P	1.48 ± 0.04	6.74 ± 0.29	7.65 ± 0.45	1.00 ± 0.01	0.87 ± 0.01
P407/C971P + mucin	0.95 ± 0.01	4.06 ± 0.18	3.32 ± 0.19	1.00 ± 0.00	0.92 ± 0.01
P407/C974P	0.64 ± 0.03	2.99 ± 0.12	2.53 ± 0.12	1.00 ± 0.01	0.91 ± 0.01
P407/C974P + mucin	0.34 ± 0.00	1.48 ± 0.08	1.20 ± 0.05	1.00 ± 0.00	1.02 ± 0.01
P407/PCB	0.56 ± 0.02	2.66 ± 0.10	2.46 ± 0.06	1.00 ± 0.00	0.92 ± 0.01
P407/PCB + mucin	0.26 ± 0.01	1.14 ± 0.06	0.93 ± 0.05	1.00 ± 0.01	0.91 ± 0.02

## Supplementary Materials

The following are available online at http://www.mdpi.com/2073-4360/10/3/254/s1, Table S1: Mucoadhesive force determined by the tensile method, on mucin disk or porcine oral mucosa, using polymeric blends containing poloxamer 407 (P407) and Carbopol 971P^®^ (C971P), Carbopol 974P^®^ (C974P), or Noveon^®^ Polycarbophil (PCB).
