# Supplementary material for: Assessing Mucoadhesion in Polymer Gels: The Effect of Method Type and Instrument Variables
Source: Polymers (Basel). 2018 Mar 1;10(3):254. doi: 10.3390/polym10030254 (PMC6415125; doi:10.3390/polym10030254)
Supplement: Supplementary file 1 [file polymers-10-00254-s001.pdf]

Supplementary Materials

Table S1. Mucoadhesive force determined by the tensile method, on mucin disk or porcine oral mucosa, using polymeric blends containing poloxamer 407 (P407) and Carbopol 971P® (C971P), Carbopol 974P® (C974P) or Noveon® Polycarbophil (PCB).

Replicate	Force (N)					
	Porcine mucin disk			Porcine oral mucosa		
	P407/C971P	P407/C974P	P407/PCB	P407/C971P	P407/C974P	P407/PCB
1	0.359	0.230	0.210	0.115	0.218	0.188
2	0.320	0.218	0.244	0.103	0.226	0.185
3	0.339	0.209	0.221	0.106	0.226	0.181
4	0.343	0.236	0.229	0.110	0.242	0.180
5	0.340	0.217	0.221	0.084	0.194	0.202
6	0.312	0.205	0.221	0.105	0.229	0.177
Mean ± SD	0.335 ± 0.017	0.219 ± 0.012	0.224 ± 0.011	0.105 ± 0.010	0.226 ± 0.016	0.183 ± 0.009

SD = standard deviation
